# The neuron-astrocyte-microglia triad in a rat model of chronic cerebral hypoperfusion: protective effect of dipyridamole

**DOI:** 10.3389/fnagi.2014.00322

**Published:** 2014-11-27

**Authors:** Daniele Lana, Alessia Melani, Anna Maria Pugliese, Sara Cipriani, Daniele Nosi, Felicita Pedata, Maria Grazia Giovannini

**Affiliations:** ^1^Section of Pharmacology and Clinical Oncology, Department of Health Sciences, University of FlorenceFlorence, Italy; ^2^Section of Pharmacology and Toxicology, Department of NEUROFARBA, University of FlorenceFlorence, Italy; ^3^Inserm U1141Paris, France; ^4^Department of Experimental and Clinical Medicine, University of FlorenceFlorence, Italy

**Keywords:** apoptosis, phagocytosis, CA1, confocal microscopy, neuron-astrocyte-microglia triad

## Abstract

Chronic cerebral hypoperfusion during aging may cause progressive neurodegeneration as ischemic conditions persist. Proper functioning of the interplay between neurons and glia is fundamental for the functional organization of the brain. The aim of our research was to study the pathophysiological mechanisms, and particularly the derangement of the interplay between neurons and astrocytes-microglia with the formation of “triads,” in a model of chronic cerebral hypoperfusion induced by the two-vessel occlusion (2VO) in adult Wistar rats (*n* = 15). The protective effect of dipyridamole given during the early phases after 2VO (4 mg/kg/day i.v., the first 7 days after 2VO) was verified (*n* = 15). Sham-operated rats (*n* = 15) were used as controls. Immunofluorescent triple staining of neurons (NeuN), astrocytes (GFAP), and microglia (IBA1) was performed 90 days after 2VO. We found significantly higher amount of “ectopic” neurons, neuronal debris and apoptotic neurons in CA1 Str. Radiatum and Str. Pyramidale of 2VO rats. In CA1 Str. Radiatum of 2VO rats the amount of astrocytes (cells/mm^2^) did not increase. In some instances several astrocytes surrounded ectopic neurons and formed a “micro scar” around them. Astrocyte branches could infiltrate the cell body of ectopic neurons, and, together with activated microglia cells formed the “triads.” In the triad, significantly more numerous in CA1 Str. Radiatum of 2VO than in sham rats, astrocytes and microglia cooperated in the phagocytosis of ectopic neurons. These events might be common mechanisms underlying many neurodegenerative processes. The frequency to which they appear might depend upon, or might be the cause of, the burden and severity of neurodegeneration. Dypiridamole significantly reverted all the above described events. The protective effect of chronic administration of dipyridamole might be a consequence of its vasodilatory, antioxidant and anti-inflammatory role during the early phases after 2VO.

## Introduction

Chronic ischemia is a progressive neurodegenerative process caused by cerebral hypoperfusion that may manifest with cognitive dysfunction as ischemic conditions persist (Sarti et al., 2002b; Schmidt-Kastner et al., 2005; Melani et al., 2010). Chronic hypoperfusion of the brain, secondary to vascular pathology, is a prominent risk factor for neurodegenerative diseases (Naritomi, 1991) such as vascular dementia (Chmayssani et al., 2007). Age-related cerebrovascular stenosis caused by arteriosclerosis induces failure of brain circulation, and resolution of cerebral hypoperfusion has been reported to improve mental decline in humans (Hirono et al., 1993; Nencini et al., 1993; Tsuda et al., 1994; Tatemichi et al., 1995).

The reconstruction of a pathological condition in animal models is a suitable approach to the unraveling of the pathophysiological mechanisms underlying neuronal damage. For the reproduction of chronic cerebral hypoperfusion as it occurs in human aging, the permanent, bilateral occlusion of the common carotid arteries in rats (two-vessel occlusion, 2VO) is a widely recognized procedure that allows to investigate the mechanisms that link chronic cerebral hypoperfusion to neurodegenerative processes (Sarti et al., 2002a,b; Farkas et al., 2007). Over the years, the 2VO model has generated a large amount of data, revealing that hippocampal neurons have higher sensitivity to ischemic insults compared to other cell populations. Specifically, the CA1 region of the hippocampus is particularly vulnerable to ischemia and to the 2VO-related pattern of hypoperfusion and metabolic changes, leading to failure of neuronal signaling, and to learning and memory disturbances (Schmidt-Kastner et al., 2001; Shibata et al., 2004; Farkas et al., 2006; Ohtaki et al., 2006; Melani et al., 2010). Since few pharmacological therapies able to ameliorate neurodegeneration in patients with chronic vascular pathologies are available, a major goal is to elucidate the pathophysiological mechanisms that link chronic hypoperfusion with alterations of brain functions to find targets for new therapeutic strategies.

Although brain disease is generally attributed to malfunction or loss of neurons, it is becoming more and more evident that proper interplay between neurons-astrocytes-microglia, the so called “triad,” is fundamental for the functional organization of the brain (Barres, 2008; Allen and Barres, 2009). Impaired interplay among neurons and glia may be responsible for derangements from normal brain physiology to neurodegenerative processes (De Keyser et al., 2008; Sofroniew, 2009). Nevertheless, the precise role of microglia-astrocytes in neurodegenerative diseases is not clearly defined and different, even contrasting, aspects have to be considered. Signals produced by neurons undergoing physiological apoptosis during normal brain aging are different from signals induced during neurodegenerative diseases (Lauber et al., 2004; Takahashi et al., 2005). How apoptosis causes neurons to die is still a matter of debate, but it is believed that the main means is by triggering production of signals which induce phagocytic cells to eat the neuron (Noda et al., 2011). Therefore, if on one side microglia and astrocytes can be neuroprotective (Faulkner et al., 2004; Myer et al., 2006; Hanisch and Kettenmann, 2007; Li et al., 2008), on the other one they can be dangerous for neuronal health (Block et al., 2007).

This work was aimed at understanding the pathophysiological mechanisms underlying chronic brain ischemia. We have demonstrated, in normal brain aging and in a model of brain inflammation, that astrocytes and microglia actively collaborate in the clearance of apoptotic neurons and neuronal debris associated with apoptosis (Cerbai et al., 2012). We used the 2VO model of cerebral hypoperfusion in the rat to verify whether this phenomenon may be a common mechanism underlying different neurodegenerative processes. Astrocytes and microglia may exert either protective mechanisms to control inflammatory processes and the spread of further cellular damage to neighboring tissue or may contribute to neuronal damage along the progression of the pathological conditions. A better understanding of risks and benefits of astrocyte and microglia activation is critical to determine whether future therapeutic interventions should impair or enhance the actions of these cells.

## Materials and methods

### Animals

Male adult Wistar rats (3 months old, Harlan, Milano, Italy) were used. The animals were housed in cages with *ad libitum* food and water and were maintained on a 12 h light–12 h dark cycle in a temperature-controlled room (23 ± 1°C). Experiments were approved by the IACUC of the University of Florence and performed according to the Italian Law on Animal Welfare (DL 116/92, Italian Directive on the protection of animals used for scientific purposes, application of the EU 609/86 directive). All efforts were made to minimize animal sufferings and to use only the number of animals necessary to produce reliable scientific data.

### Surgery

**Figure 1:**
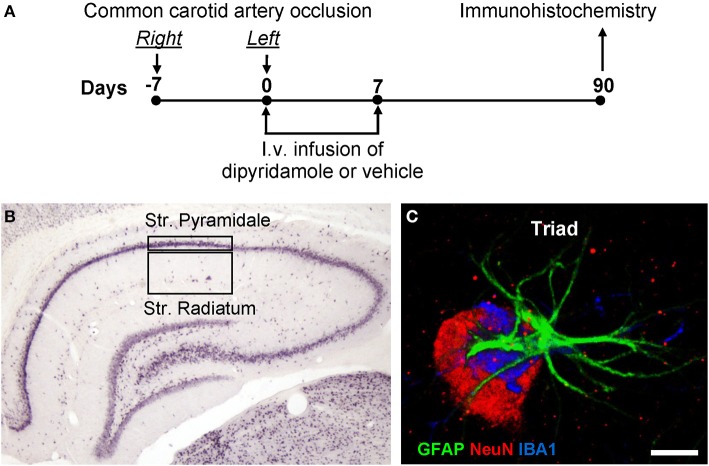
**(A)** Experimental scheme. Chronic hypoperfusion in the rat was obtained by occlusion of the Right and Left common carotid arteries separately, 7 days apart. Dipyridamole was administered i.v. for 7 days. **(B)** Representation of the regions of Interest (ROI) in CA1 hippocampus Str. Pyramidale and Str. Radiatum where all imunohistochemical analyses were performed. **(C)** Representation of a triple immunostaining of a neuron-astrocyte-microglia triad. Neuron (NeuN positive, red), astrocyte (GFAP positive, green), microglia (IBA1 positive, blue). Scale bar: 5 μm.

Bilateral common carotid artery occlusion (2VO) was carried out in rats (Sarti et al., 2002a,b; Farkas et al., 2007) as follows, according to the scheme represented in Figure 1A. Anesthesia was induced with 5% halothane and maintained with spontaneous respiration of 1/2% halothane in air by use of a face mask. Body temperature was maintained at 37°C with a recirculating pad and K module and was monitored via an intrarectal type T thermocouple (Harvard, Kent, UK). The *right* common carotid artery was occluded, (day-7, Figure 1A), as follows. The skin of the ventral part of the neck was incised and the omohyoid muscle was cut through a median incision. Using a microscope, the common carotid artery was exposed and firmly ligated with a silk suture. In sham-operated rats the same procedure was applied without the ligature. After 1 week (day 0, Figure 1A), the same procedure was repeated to occlude the *left* common carotid artery. After each surgical procedure, rats were treated daily with diaminocillin (1,200,00 U in 8 ml saline, 1 ml/day i.m.). Animals were randomly subdivided in three experimental groups: sham-operated rats (sham, *n* = 15), 2VO-operated rats treated with vehicle (2VO-vehicle, *n* = 15), and 2VO-operated rats treated with dipyridamole (2VO-dipyridamole, *n* = 15) from day 0 to day 7. We did not include the sham-dipyridamole-treated animals group in the project because we wanted to verify the effect of the drug in 2VO treated rats.

### Drug administration

Dipyridamole (Persantin, Boehringer Ingelheim, 5 mg/mL) was administered by intravenous perfusion to obtain stable concentrations of the drug in the blood. Dipyridamole or vehicle were injected (10 μL/h per 7 days) into the jugular vein by a miniosmotic pump (Model 2001, Alzet, Cupertino, CA, USA) attached to a silicone catheter (0.51 mm ID × 0.07 wall; Alzet) and prefilled with drug or vehicle in sterile conditions, followed by a 12-h preimplantation incubation at 37°C. The pumps were implanted subcutaneously in the thoraco-lumbar region immediately after the left common carotid artery occlusion and removed after 7 days (Figure 1A). Considering the body weight of rats (about 300 g), each animal received about 4 mg/kg/day of dipyridamole. On the basis of a previous estimation of dipyridamole plasma concentration in the rat, we estimated that administration of 4 mg/kg dipyridamole i.v. provides a 2–2.5 μM plasma concentration over the entire week (Newell et al., 1986). The dose of dipyridamole administered in this study is similar to that (0.5–3 mg/kg i.v.) used by Jones (Jones et al., 1994). This concentration (equivalent to 1 μg/mL) is close to the therapeutic concentration (1.6 μg/mL) reached after extended-release dipyridamole administered to ischemic stroke patients (Serebruany et al., 2009).

### Immunohistochemistry

Ninety days after surgery, rats were anesthetized (chloral hydrate, 400 mg/kg) and perfused transcardially with 500 ml of ice-cold paraformaldehyde (4% in phosphate-buffered saline, PBS, pH 7.4). Brains were postfixed for 4 h and cryoprotected in 18% sucrose/PBS for at least 48 h. Coronal sections (thickness 40 μm) were cut with a cryostat, placed in 1 ml of anti-freeze solution (40% PBS, 30% Ethylene-Glycol, 30%, Glycerol, v/v) and stored at −20°C until immunohistochemistry.

#### Antibodies

The following primary antibodies were used: a mouse monoclonal anti-neuronal nuclei (NeuN, 1:200; Millipore, Billerica, MA) for neurons; a rabbit polyclonal anti-glial fibrillary acidic protein (GFAP, 1:1000; DakoCytomation, Glostrup, Denmark) for astrocytes; a rabbit polyclonal anti-IBA1 (1:300, WAKO Pure Chem. Ind., Osaka, Japan) for microglia; a mouse monoclonal anti-Cytochrome C (Cyt C, 1:200, Becton and Dickinson, Franklin Lakes, NJ, USA) as an apoptotic marker. In triple staining for astrocytes, microglia and neurons, astrocytes were labeled with a mouse monoclonal anti-GFAP antibody conjugated with the fluorochrome AlexaFluor 488 (1:500), in triple staining for neurons, microglia and cytochrome C, neurons were labeled with a mouse monoclonal anti-NeuN antibody conjugated with the fluorochrome AlexaFluor 488 (1:500), both from Millipore, Billerica, MA.

The following fluorescent secondary antibodies were used: AlexaFluor 488 donkey anti-rabbit (1:400), AlexaFluor 594 goat anti-mouse (1:400), AlexaFluor 555 donkey anti-mouse (1:400), Alexa Fluor 635 goat anti-rabbit (1:400) (all from Life Technologies, Carlsbad, CA, USA). Colocalization of different antigens was performed using combinations of different primary and secondary antibodies, using double or triple labeling confocal microscopy. Nuclei were stained using DAPI (Vectashield hard set mounting medium containing DAPI, Vector Laboratories).

#### Fluorescent immunohistochemistry

Immunostaining was performed on coronal slices with the free-floating method (Giovannini, 2002). Day 1. Free-floating slices (40 μm thick) were placed in wells, were rinsed three times for 5 min in PBS-TX and then were blocked for 60 min with BB containing 10% Normal Goat Serum–10% Normal Horse Serum in PBS-TX and 0.05% NaN_3_. Slices were incubated overnight at 4°C under slight agitation with a combination of two different primary antibodies dissolved in BB (depending on the antigens we wanted to visualize).

Day 2. For double immunostaining, slices were incubated for 2 h at room temperature in the dark with the appropriate secondary antibody (see above).

For triple immunostaining, slices were incubated for 2 h at room temperature in the dark with AlexaFluor 555 donkey anti mouse IgG (1:400) secondary antibody and then for 2 h at room temperature in the dark with AlexaFluor 555 donkey anti mouse IgG (1:400) plus Alexa Fluor 635 goat anti-rabbit (1:400). Depending on the antigens to be visualized, after 3 washings, astrocytes were immunostained using the primary antibody anti-GFAP AlexaFluor 488-conjugated and neurons were immunostained using the primary antibody anti-NeuN AlexaFluor 488-conjugated. For both double and triple immunostaining procedures, slices were mounted onto gelatin-coated slides using Vectashield hard set mounting medium with DAPI (Vector Laboratories).

#### Widefield and confocal fluorescence microscopy

Slices were observed under an epifluorescent microscope Olympus BX40 equipped with an Olympus DP 50 digital camera (Olympus, Milan, Italy) or under a LEICA TCS SP5 confocal laser scanning microscope (Leica Microsystems CMS GmbH, Mannheim, Germany). Confocal scans were taken at 0.3 μm z-steps keeping pinhole, contrast and brightness constant. Voxel size was 6.75 × 10^−3^ μm^3^. Images were digitally converted to green, red or blue using Image J (National Institute of Health, http://rsb.info.nih.gov/ij), digitally combined to obtain single, double or triple labeled images and then assembled into montages. High power 3D renderings of the images were obtained using Image J 3D viewer.

### Methodological considerations

All quantification analyses were performed by two researchers blind to the experimental conditions and results were averaged. Three coronal slices (spaced by 150 μm, starting at about −2.8 mm from bregma) containing the dorsal hippocampi were immunostained. Since no differences was ever found between the two hippocampi (Sarti et al., 2002b and unpublished results) the regions of interest (ROI) of both CA1 areas, containing Str. Pyramidale or Str. Radiatum were consistently captured at 20× magnification using an Olympus digital camera (framed area in Figure 1B). Areas of ROI in CA1 were calculated in mm^2^. Astrocytes, microglia, ectopic neurons, neuronal debris, and the triads (neurons-astrocytes-microglia) in CA1 Str. Radiatum, and apoptotic neurons and DAPI positive cells in CA1 Str. Pyramidale were consistently counted in the same area in all slices and were expressed as cells/mm^2^. DAPI stained cells constitute the total cell population in CA1 Str. Pyramidale (neurons plus astrocytes plus microglia). Subtracting the number of GFAP positive astrocytes and IBA1 positive microglia in Str. Pyramidale from total DAPI stained cells it is possible to obtain the number of neurons in CA1 Str. Pyramidale. Therefore, quantitation of CA1 pyramidal neurons was obtained subtracting the number of astrocytes and microglia cells (counted separately) from the total of DAPI positive cells in CA1 Str. Pyramidale. We defined “triad” a neuron that was in direct contact with a microglia cell and astrocyte branches deriving from surrounding astrocyte(s), as exemplified by the confocal 3D rendering shown in Figure 1C. Cell debris was defined as NeuN-positive fragments with dimensions between 2.5 and 6.5 μm. NeuN immunostaning labels a protein that is located not only in the nuclei, but also in the surrounding cytoplasm and in the proxymal parts of dendrites, as visible from images present in our and others' work (Cobos et al., 2005; Cerbai et al., 2012). Therefore, it is plausible that debris are NeuN positive. The criteria to characterize a microglia cell as “resting” or “reactive” were defined before evaluation as follows. In accordance to the literature (Stence et al., 2001; Nelson et al., 2002; Rezaie et al., 2002; Herber et al., 2006; Miller and Streit, 2007) resting microglia were defined as cells with small, round cell bodies with thin and highly ramified branches equally distributed around the cell body. Reactive microglia were defined as cells with a large pleomorphic bi-or tri-polar cell body, or as spindle or rod-shaped cells with modification in cellular structure that included de-ramification and shortening and twisting of cellular processes (Cerbai et al., 2012). In order to unambiguously define whether astrocyte branches were infiltrating neurons we digitally cut the 3D rendering of the cell along a vertical plane and rotated the image around the cell vertical axis by 45 and 90°. Confocal qualitative double or triple immunostaining analyses were performed in 2VO-vehicle (*n* = 6), 2VO-dipyridamole (*n* = 6), and sham rats (*n* = 6). To verify the specificity of the staining, control immunostaining was performed omitting the primary or secondary antibodies.

### Statistical analysis

Statistical comparisons were performed using Graph Pad Prism (Graph Pad Software Inc., La Jolla, CA, USA) by One-Way ANOVA followed by Newman–Keuls Multiple Comparison Test, or Two-Way ANOVA followed by Bonferroni multiple comparison test, as appropriate. Significance was set at *P* < 0.05.

## Results

We previously demonstrated in two different animal models of neurodegeneration that astrocytes and microglia appear to collaborate in the clearance of apoptotic neurons and neuronal debris in the CA1 region of the hippocampus (Cerbai et al., 2012). Using the novel method of simultaneous triple immunostaining of neurons, astrocytes and microglia, we further characterized the interplay among neurons and glia in this animal model of chronic cerebral hypoperfusion and neurodegeneration.

### “Ectopic” neurons and neuronal debris in the CA1 Str. Radiatum

**Figure 2:**
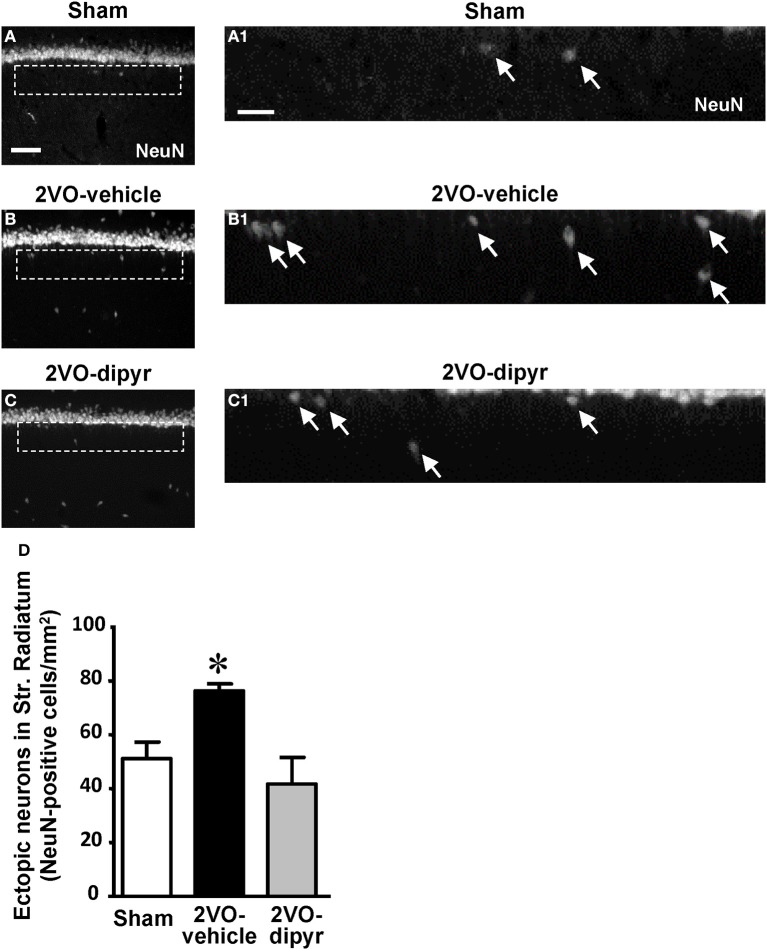
**Quantitative analysis of ectopic neurons in CA1 Str. Radiatum. (A–C)** Representative photomicrographs of immunostained neurons (NeuN positive), taken at an epifluorescent microscope, in CA1 Str. Pyramidale and CA1 Str. Radiatum of a sham **(A)**, a 2VO-vehicle **(B)**, and a 2VO-dipyridamole rat **(C)**. Images show the presence of ectopic neurons in the Str. Radiatum within 100 μm from the Str. Pyramidale. Scale bar: 70 μm. **(A1–C1)** Higher magnification images of the framed areas shown in **(A–C)**, respectively. Arrows indicate ectopic neurons. Scale bar: 25 μm. **(D)** Quantitative analysis of ectopic neurons in CA1 Str. Radiatum of sham (white column, *n* = 7), 2VO-vehicle (black column, *n* = 8), and 2VO-dipyridamole (gray column, *n* = 8) rats (ectopic neurons/mm^2^; mean ± s.e.m.; ^*^at least *P* < 0.05 vs. sham and 2VO-dipyridamole rats, One-Way ANOVA followed by Newman–Keuls Multiple Comparison Test). Quantification was performed blind by two researchers in the region of interest (ROI) of CA1 Str. Radiatum and results were averaged. ROIs were calculated in mm^2^ and the counts of NeuN immunopositive cells were expressed as neurons/mm^2^.

Immunostaining of neurons using the anti-NeuN antibody revealed the presence of numerous neurons, that we defined “*ectopic*,” scattered throughout the CA1 Str. Radiatum of sham (Figures 2A,A1), 2VO-vehicle (Figures 2B,B1), and 2VO-dipyridamole rats (Figures 2C,C1). Ectopic neurons were mostly present in the Str. Radiatum within 100 μm from the Str. Pyramidale, (arrows in Figures 2A1–C1, magnifications of the framed areas shown in Figures 2A–C). Quantitative analysis performed in the Str. Radiatum showed that ectopic neurons were significantly more numerous (+49%) in the CA1 Str. Radiatum of 2VO-vehicle rats than in sham operated rats. Dipyridamole completely reverted this phenomenon [Figure 2D, One-Way ANOVA *F*_(2, 20)_ = 6.844; *P* = 0.0054; ^*^at least *P* < 0.05 vs. both other groups, Newman–Keuls Multiple Comparison Test].

**Figure 3:**
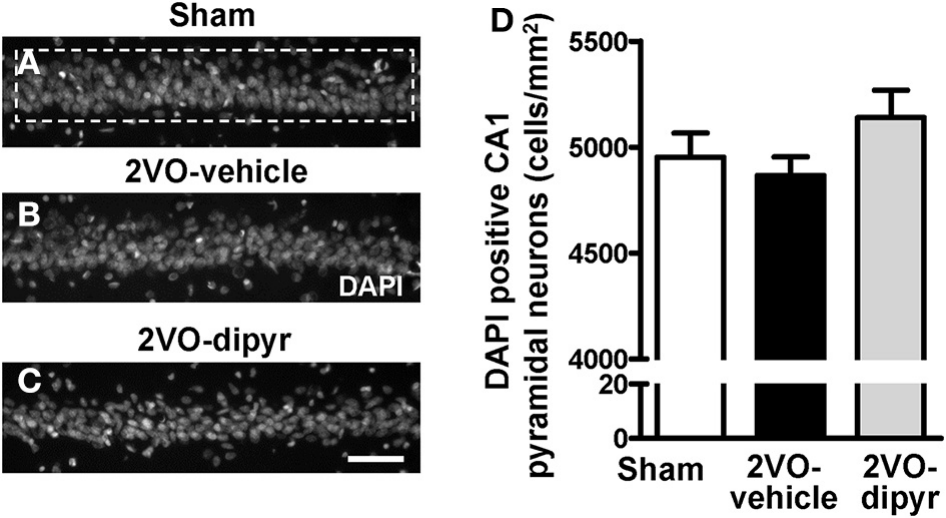
**Quantitative analysis of cells labeled with DAPI in CA1 Str. Pyramidale. (A–C)** Representative photomicrographs, taken at an epifluorescent microscope, of DAPI staining in CA1 Str. Pyramidale of a sham **(A)**, a 2VO-vehicle **(B)**, and a 2VO-dipyridamole rat **(C)**. Scale bar: 50 μm. **(D)** Quantitative analysis of DAPI positive pyramidal neurons in CA1 Str. Pyramidale of sham (white column, *n* = 12), 2VO-vehicle (black column, *n* = 12), and 2VO-dipyridamole (gray column, *n* = 11) rats (DAPI positive neurons/mm^2^; mean ± s.e.m.; not significant, One-Way ANOVA). Quantification of CA1 pyramidal neurons was obtained subtracting the quantity of GFAP positive astrocytes and IBA1 positive microglia from the total of DAPI positive cells in CA1 ROI (area highlighted by the dashed rectangle in **A**). Quantification was performed blind by two researchers in the region of interest (ROI) of CA1 Str. Pyramidale (framed area in **A**) and results were averaged. ROIs were calculated in mm^2^ and the counts of DAPI positive neurons were expressed as cells/mm^2^.

We evaluated whether chronic hypoperfusion might cause loss of CA1 pyramidal neurons. Cells were counted in the ROI of sham (Figure 3A), 2VO-vehicle (Figure 3B), and 2VO-dipyridamole rats (Figure 3C) (framed area in Figure 3A; for the procedure see Methodological considerations). Results in Figure 3D show that the amounts of CA1 pyramidal neurons (cells/mm^2^) were not significantly different among the three experimental groups [One-Way ANOVA *F*_(2, 32)_ = 1.56; *P* = 0.226, n.s.].

### Quantification of astrocytes in the CA1 Str. Pyramidale and in CA1 Str. Radiatum

In order to verify the hypothesis that astrocytes and microglia might actively collaborate in the triad formation around apoptotic neurons and neuronal debris in the CA1 region of the hippocampus of 2VO rats (Cerbai et al., 2012), we studied the interplay among neurons and microglia using the triple immunostaining of neurons, astrocytes, and microglia in the CA1 Str. Pyramidale and CA1 Str. Radiatum of sham, 2VO-vehicle and 2VO-dipyridamole rats.

**Figure 4:**
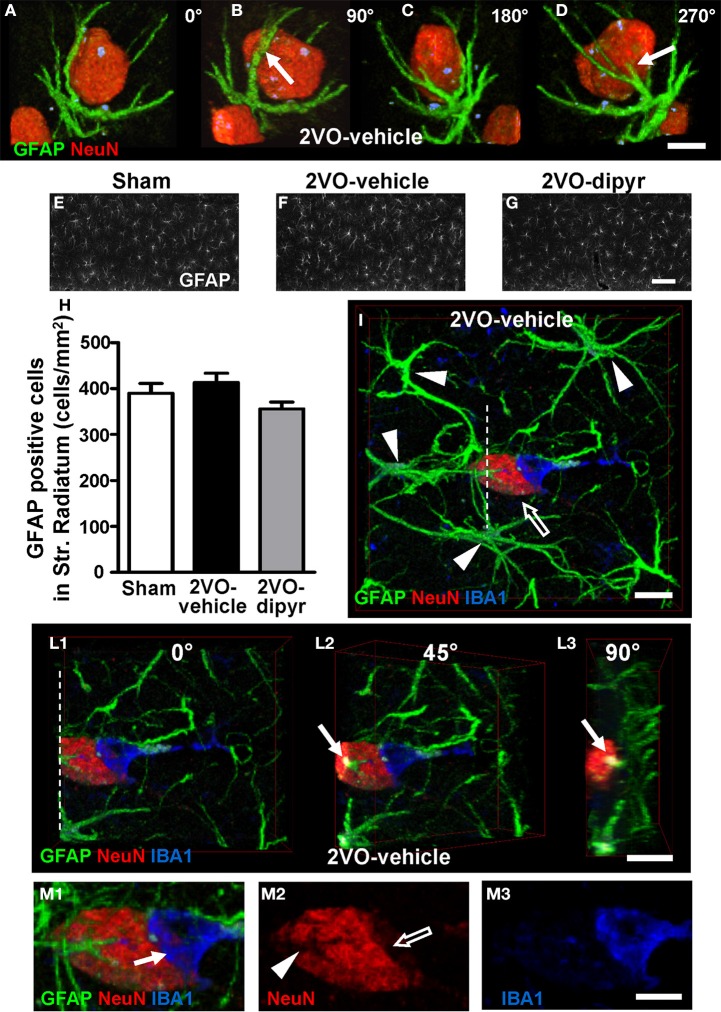
**Neuron-astrocyte-microglia interplay and quantification of astrocytes. (A–D)** Double immunostaining of neurons (red) and astrocytes (green). The confocal images represent 3D renderings of 49 confocal scans (total thickness 14.7 μm) acquired starting at 0.3 μm depth into the slice. The confocal image shown in **(A)** was digitally rotated by 90° **(B)**, 180° **(C)**, and 270° **(D)** around its vertical axis. Scale bar: 7.5 μm. (**E–G)** Representative epifluorescent photomicrographs of GFAP immunostained astrocytes in CA1 Str. Radiatum of a sham **(E)**, a 2VO-vehicle **(F)**, and a 2VO-dipyridamole rat **(G)**. GFAP positive astrocytes were counted in Str. Radiatum (ROI as represented in **E–G**). Scale bar: 50 μm. **(H)** Quantitative analysis of GFAP positive astrocytes in CA1 Str. Radiatum of sham (white column, *n* = 11), 2VO-vehicle (black column, *n* = 8), and 2VO-dipyridamole rats (gray column, *n* = 10) counted as described above (GFAP positive cells/mm^2^; mean ± s.e.m.; n.s., One-Way ANOVA). Quantification was performed blind by two researchers and results were averaged. ROIs were calculated in mm^2^ and the counts of astrocytes were expressed as neurons/mm^2^. **(I–L3)** Representative confocal images of triple immunostaining of astrocytes (green), neurons (red, open arrow), and microglia (blue) in the CA1 Str. Radiatum of a 2VO-vehicle rat. The 3D confocal rendering in **(I)** was obtained from 30 confocal scans (total thickness 9.0 μm) acquired starting at 3 μm depth into the slice. Arrowheads show the bodies of 4 astrocytes projecting their branches toward the neuron and forming a glia “micro scar” around it. The 3D confocal rendering in **(I)** was digitally cut along the white dotted line **(L1)** and rotated by 45° **(L2)** and 90° **(L3)** around the vertical axis. Arrows in **(L2, L3)** show that an astrocyte branch infiltrates the neuronal cell body. Scale bar in **I–L3**: 10 μm. (**M1–M3)** High magnification confocal renderings of the neuron-astrocyte-microglia triad shown in **(I)**. Arrow in **(M1)** indicates an IBA1-positive microglia cell phagocytosing the neuron. Panel **(M2)** shows a neuron immunostained for NeuN. The open arrow indicates that neuronal cytoplasm is missing underneath the phagocitosing microglia cell. Arrowhead shows the grooves formed by astrocyte branches infiltrating the neuron cell body. Panel **(M3)** shows the IBA1 positive amoeboid microglia cell. Scale bar: 5 μm.

Confocal images in Figures 4A–D show the close interplay between an ectopic neuron immunostained with anti-NeuN antibody (red) and an astrocyte with anti-GFAP antibody (green) in the CA1 Str. Radiatum of a 2VO-vehicle rat. To better visualize this phenomenon the cell confocal 3D rendering of the neuron shown in Figure 4A was digitally rotated by 90° (Figure 4B), 180° (Figure 4C), and 270° (Figure 4D) around its vertical axis. It is evident from the images that astrocyte branches (arrows) are in intimate relationship with the neuronal cell body (see also Supporting Information “Media File 1”). Indeed, in the CA1 Str. Radiatum of 2VO-vehicle rats ectopic neurons were often surrounded by astrocyte branches, which were in close proximity to the cell body, embracing and apparently wedging them.

Astrocytes were quantified in the ROI of Str. Radiatum, as shown in the representative images of sham (Figure 4E), 2VO-vehicle (Figure 4F), and 2VO-dipyridamole rats (Figure 4G). Quantitative analysis (Figure 4H) showed that GFAP positive cells did not change significantly in CA1 Str. Radiatum of 2VO-vehicle rats in comparison to the other experimental groups [One-Way ANOVA *F*_(2, 26)_ = 2.135; *P* = 0.1385, n.s.].

Neurons, astrocytes, and microglia were triple immunostained in the CA1 of sham (*n* = 6), 2VO-vehicle (*n* = 6), and 2VO-dipyridamole (*n* = 6) rats. Triple immunostaining of astrocytes with anti-GFAP antibody (green), neurons with anti-NeuN antibody (red), and microglia with anti-IBA1 antibody (blue) in the CA1 Str. Radiatum of a 2VO-vehicle rat clearly show the presence of a neuron-astrocytes- microglia triad (Figure 4I). It is clearly visible from the image that an ectopic neuron is surrounded by astrocyte branches deriving from at least 4 different astrocytes (arrowheads) that form a scar around it. Although not more numerous, astrocytes appeared to be more active in the CA1 Str. Radiatum of 2VO-vehicle rats in comparison to sham and 2VO-dipyridamole animals.

The confocal 3D rendering of the neuron shown in Figure 4I was digitally cut along the dotted white line (Figure 4L1), and the 3D rendering was digitally rotated by 45° (Figure 4L2) and 90° (Figure 4L3) around its vertical axis to visualize the inside of the neuronal cell body. It is clearly visible from Figures 4L2,L3 (arrows) that an astrocyte branch infiltrates the neuronal cell body. These morphological features are consistent with the hypothesis that astrocytes are bisecting a ectopic neuron to form neuronal debris (further proof is provided below in the next paragraphs).

To complete the triad is the presence of a microglia cell (IBA1 positive, blue) in close contact with the neuronal cell body and possibly phagocytosing the neuron. Indeed, it is clearly visible from the magnifications in Figures 4M1–M3 that a reactive microglia cell, which has lost most of its branches and has acquired an almost amoeboid shape typical of phagocytic microglia (Beynon and Walker, 2012), wraps and phagocytoses the neuron (arrow in Figure 4M1). Portion of the neuronal cytoplasm is clearly missing (open arrow in Figure 4M2). Figure 4M2 also clearly shows the grooves formed by the astrocyte branches infiltrating the neuronal cytoplasm (arrowhead), which correspond to the astrocyte branches that penetrate the neuronal cell body (pointed out by the arrows in Figures 4L2,L3). Triple immunostaining confocal 3D renderings were taken from shams (*n* = 4), 2VO-vehicle (*n* = 5), and 2VO-dipyridamole rats (*n* = 4) and the neuron-astrocytes-microglia triads which were often found in 2VO-vehicle rats, were more rarely observed in sham and 2VO-dypiridamole rats (see also **Figure 8D**).

### Quantification of neuronal debris in CA1 Str. Radiatum

**Figure 5:**
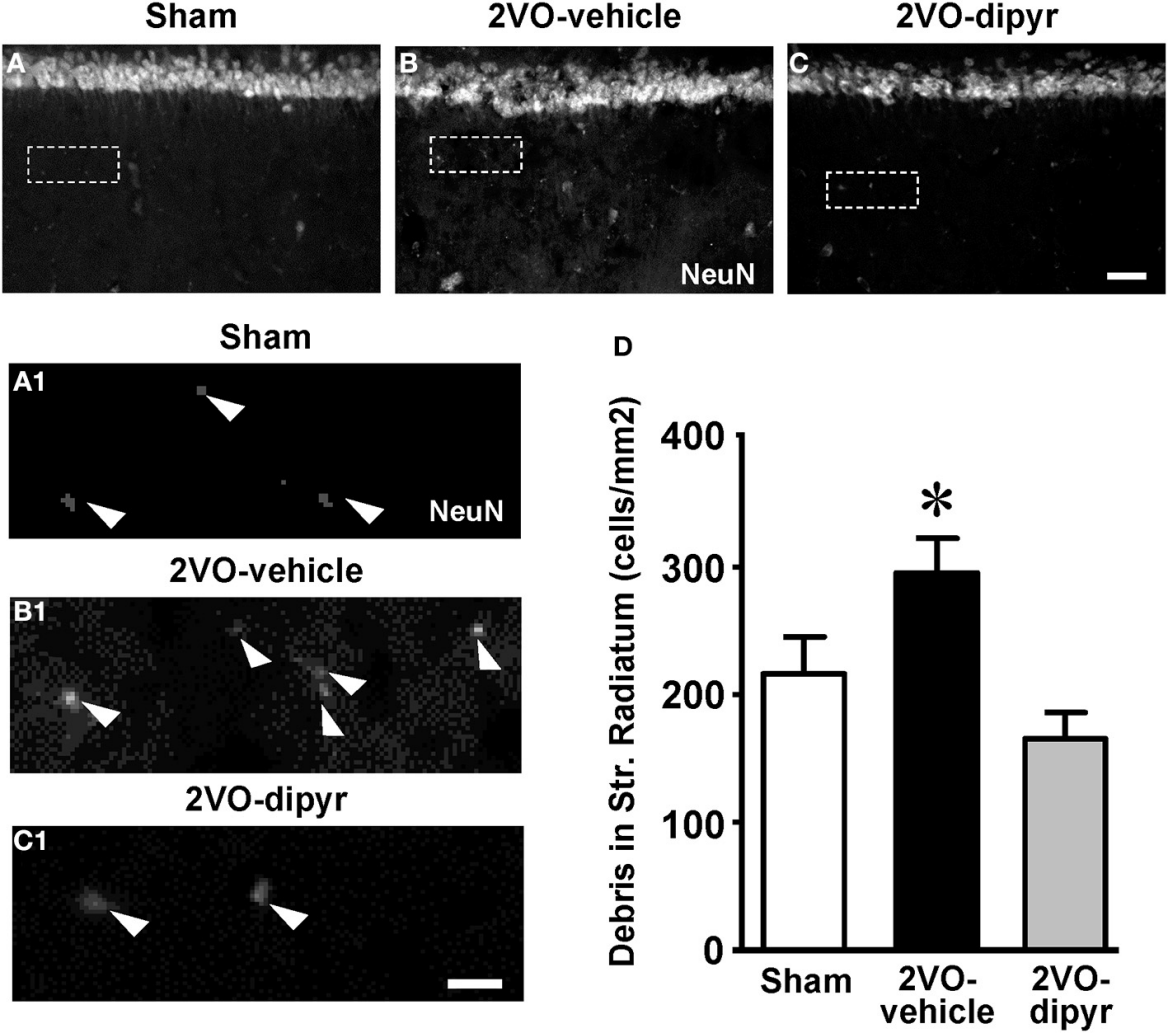
**Quantitative analysis of neuronal debris in CA1 Str. Radiatum. (A–C)** Representative photomicrographs, taken at an epifluorescent microscope, of NeuN immunostaining in CA1 Str. Pyramidale and CA1 Str. Radiatum of a sham **(A)**, a 2VO-vehicle **(B)**, and a 2VO-dipyridamole rat **(C)** showing the presence of NeuN positive debris in the Str. Radiatum (arrowheads in the magnifications **A1–C1**). Neuronal debris were defined as NeuN-positive fragments with dimensions ranging between 2.5 and 6.5 μm. Scale bar: 75 μm. **(A1–C1)** High magnification images of the framed areas shown in (**A–C)**. Scale bar: 10 μm. **(D)** Quantitative analysis of neuronal debris in CA1 Str. Radiatum of sham (white column, *n* = 9), 2VO-vehicle (black column, *n* = 10), and 2VO-dipyridamole rats (gray column, *n* = 11) counted in CA1 Str. Radiatum (debris/mm^2^; mean ± s.e.m.; ^*^at least *P* < 0.05 vs. sham and vs. 2VO-dipyridamole rats; One-Way ANOVA and Newman–Keuls Multiple Comparison Test). Quantification was performed blind by two researchers in the region of interest (ROI) of CA1 Str. Radiatum and results were averaged. ROIs were calculated in mm^2^ and the counts of NeuN positive debris were expressed as neurons/mm^2^.

As already demonstrated in aged and LPS-treated rats (Cerbai et al., 2012), astrocytes branches infiltrating the neuronal cell body seem to be fragmenting ectopic neurons to form debris, thus cooperating with the microglia in phagocytic events. NeuN staining revealed the presence of neuronal debris (NeuN positive fragments 2.5–6.5 μm in diameter) scattered throughout the CA1 Str. Radiatum of sham, 2VO-vehicle and 2VO-dipyridamole rats (arrowheads in Figures 5A1–C1). Quantitative analysis in the ROI of Str. Radiatum (framed areas in Figures 5A–C) showed that neuronal debris were significantly more numerous in the CA1 Str. Radiatum of 2VO-vehicle rats (+37%) than in sham rats (Figure 5D). Dypiridamole completely reverted this effect [One-Way ANOVA *F*_(2, 27)_ = 6.752; *P* = 0.0042; ^*^at least *P* < 0.05 vs. two other groups, Newman–Keuls Multiple Comparison Test].

### Quantification of microglia in CA1 Str. Radiatum

**Figure 6:**
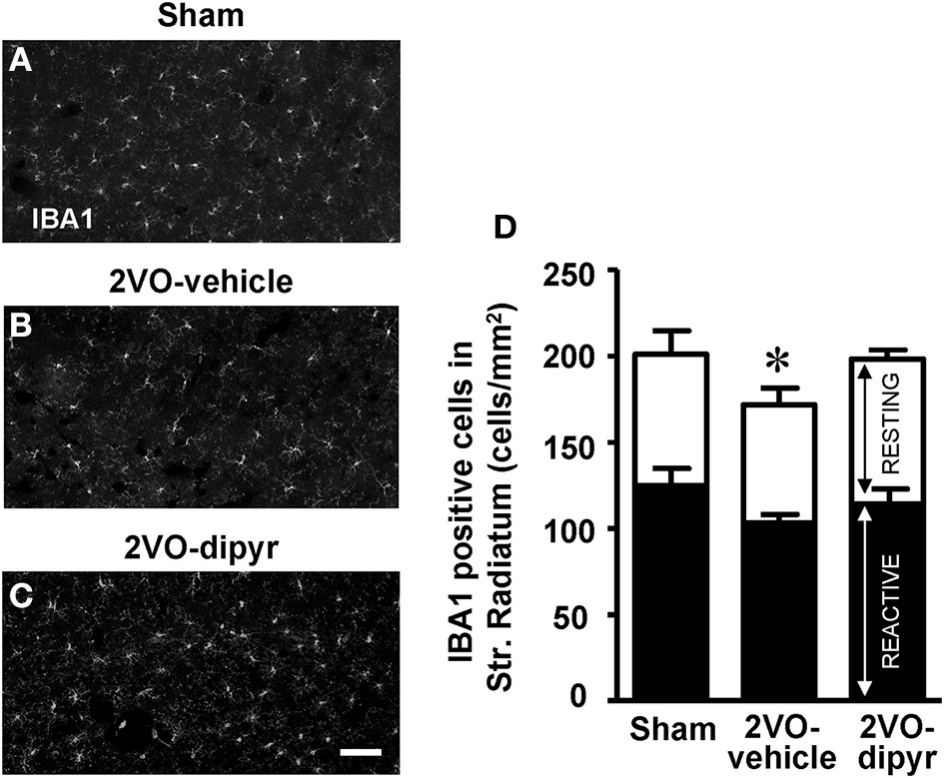
**Quantitative analysis of resting and reactive microglia cells. (A–C)** Representative photomicrographs, taken at an epifluorescent microscope, of IBA1 positive cells in CA1 Str. Radiatum of a sham **(A)**, a 2VO-vehicle **(B)**, and a 2VO-dipyridamole rat **(C)**. Scale bar: 50 μm. **(D)** Quantitative analysis of microglia cells in CA1 Str. Radiatum of sham (*n* = 8), 2VO-vehicle (*n* = 8), and 2VO-dipyridamole rats (*n* = 11). Black portion of columns represent resting microglia cells, white portion of columns represent reactive microglia cells, the entire columns represent total microglia cells. Total microglia in 2VO-vehicle rats was significantly different from sham and 2VO-dipyridamole rats (IBA1 positive cells/mm^2^; mean ± s.e.m.; ^*^P < 0.05 vs. the two other groups, One-Way ANOVA and Newman–Keuls Multiple Comparison Test). Resting and reactive microglia did not differ among the three experimental groups. Quantification was performed blind by two researchers in the region of interest (ROI) of CA1 Str. Radiatum (represented in **A–C**) and results were averaged. ROIs were calculated in mm^2^ and the counts of resting and reactive microglia were expressed as neurons/mm^2^.

We performed immunostaining of microglia (using the anti IBA1 antibody) in the CA1 Str. Radiatum of the three experimental groups. IBA1 positive resting and reactive microglia cells, characterized as described in the Materials and Methods Section, were quantified in the ROI of Str. Radiatum (Figures 6A–C). Each column in Figure 6D, subdivided into reactive microglia (white) and resting microglia (black), represents total microglia (cells/mm^2^) in the CA1 Str. Radiatum of the three experimental groups. In 2VO-vehicle rats, both resting and reactive microglia decreased slightly in comparison to shams (−9 and −14%, respectively), but these differences were not statistically significant (One-Way ANOVA, n.s.). Total microglia cells slightly but significantly decreased in CA1 Str. Radiatum of 2VO-vehicle rats [−18% vs. sham rats, One-Way ANOVA, *F*_(2, 23)_ = 4.194; *P* = 0.028; ^*^P < 0.05 vs. two other groups, Newman–Keuls Multiple Comparison Test], and dipyridamole reverted this effect (−2% vs. sham, n.s.).

### Characterization and quantification of neuron-astrocytes-microglia triads in CA1 Str. Radiatum

**Figure 7:**
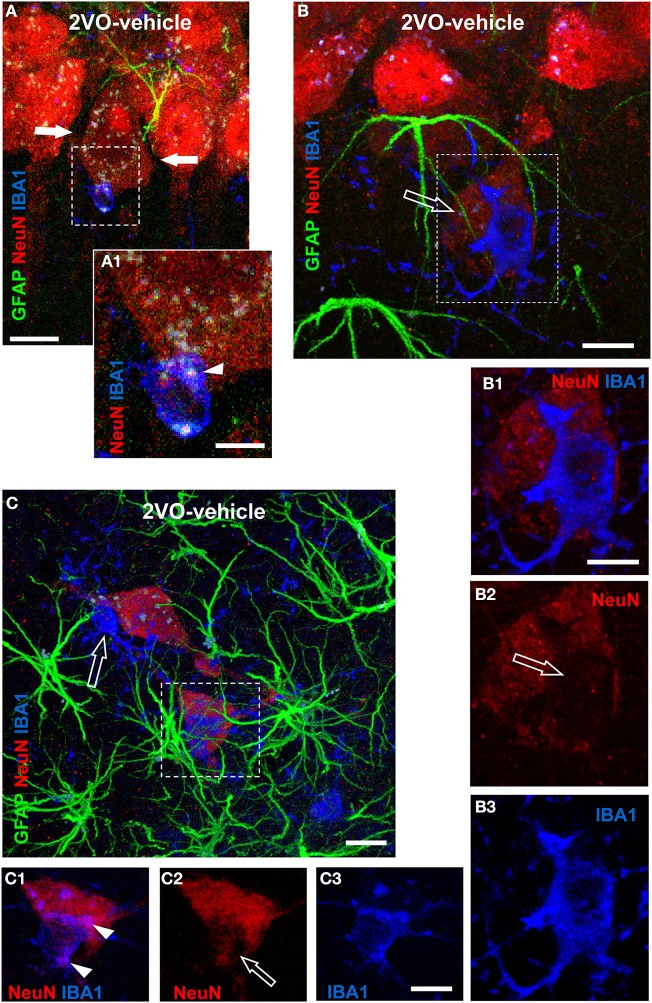
**Evidence of triads and phagocytosis of neurons in CA1 Str. Radiatum of 2VO-vehicle rats**. Triple immunostaining of GFAP (green), NeuN (red), and IBA1 (blue) in CA1 Str. Pyramidale and Str. Radiatum of three different 2VO-vehicle rats. **(A)** Confocal 3D rendering of triple immunostaining in the CA1 Str. Pyramidale and Str. Radiatum of a 2VO-vehicle rat obtained stacking three consecutive confocal z scans (0.3 μm each, total thickness 0.9 μm acquired at 2.8 μm depth into the neuron). Arrows indicate an empty space that separates the neuron from the surrounding CA1 pyramidal neurons. Scale bar: 5 μm. **(A1)** Magnification of the framed area in **(A)**. The arrowhead shows that the neuronal cytoplasm is inside the microglia cell body. Scale bar: 2 μm. **(B)** Confocal 3D rendering of triple immunostaining in the CA1 Str. Pyramidale and Str. Radiatum of a different 2VO-vehicle rat. The image represents a 3D rendering of 33 confocal scans (total thickness 9.9 μm) acquired starting at 0.3 μm depth into the slice. The ectopic neuron (open arrow) is in close contact with both astrocyte branches and a microglia cell that resides on top of the neuron and embraces it with its branches. Scale bar: 10 μm. **(B1–B3)** Magnifications of the framed area in **(B)**. Astrocytes were omitted for clarity. The open arrow in **(B2)** shows the lack of NeuN immunostaining beneath the microglia cell, indicating that the microglia is phagocytosing the neuron. Scale bar: 10 μm. **(C)** Triple immunostaining in the CA1 Str. Radiatum of a third 2VO-vehicle rat. This image represents a 3D rendering of 37 confocal scans (total thickness 11.1 μm) acquired starting at 3.6 μm depth into the slice. Two ectopic neurons (red) are surrounded by astrocyte branches and phagocytosed by microglia cells. The open arrow shows a fusiform-shaped microglia cell. Scale bar: 8 μm. (**C1–C3)** Higher magnification confocal “sub-slices” of the framed area in **(C)** obtained stacking 12 consecutive confocal z scans (0.3 μm each, total thickness 3.6 μm acquired at 1.5 μm depth into the neuron). Astrocytes were omitted for clarity. Arrowheads in **(C1)** indicate colocalization of neuronal cytoplasm within IBA1-positive reactive microglia cell. Open arrow in **(C2)** indicates loss of NeuN staining. Scale bar: 5 μm.

Although microglia cells in the CA1 Str. Radiatum of 2VO-vehicle rats were less numerous than in sham rats, microglia was still actively involved in phagocytosis of neurons, as shown in the representative confocal images presented in Figure 7, taken from three independent 2VO-vehicle rats. Indeed, the confocal 3D renderings (Figures 7A–C), show that astrocytes (green) and microglia (blue) are in close interactions with neurons (red), forming triads. Figure 7A, taken from a 2VO-vehicle rat shows that a microglia cell (blue), which has an almost amoeboid shape, is in close contact with a neuronal cell body in the CA1 pyramidal cell layer. Figure 7A1 represents a magnification of the framed area in Figure 7A. It is clearly visible that part of the neuronal cell body is internalized within the microglia cytoplasm (Figure 7A1, arrowhead). It is also apparent from Figure 7A that this pyramidal neuron seems to be in the process of detaching from the pyramidal cell layer. Indeed, an empty space, evidenced by the two arrows, is forming around the neuron in the pyramidal cell layer.

Figure 7B shows a confocal 3D rendering of an immunostained hippocampal slice taken from a different 2VO-vehicle rat. The image shows that a neuron (open arrow), located within 20 μm from the CA1 Str. Pyramidale, is in close contact with both astrocyte branches and a microglia cell. Panels **7B1–B3** represent magnifications of the framed area of Figure 7B and clearly show that a reactive microglia cell, with an enlarged cell body and thick and short branches, is in close contact with the neuronal cell body and is starting to phagocytose it (see the open arrow in Figure 7B2, indicating the missing cytoplasm under the microglia cell).

Figure 7C, taken in the Str. Radiatum of a third 2VO-vehicle rat, shows that two ectopic neurons are embraced by astrocyte branches deriving from different astrocytes and forming a “micro scar” around them, as also demonstrated in Figure 4I. The two ectopic neurons are in close contact with reactive microglia cells that are phagocytosing them (see below). The microglia cell pointed out by the open arrow has a fusiform shape, while the other one has an almost amoeboid shape, both typical of reactive microglia (Beynon and Walker, 2012). Magnifications of the framed area in Figure 7C are shown in panels **7C1–C3**. Astrocytes were omitted for clarity. Again, the images clearly show that a reactive, almost amoeboid microglia cells is phagocytosing the neuron, since part of the neuronal cytoplasm is internalized within the microglia cell (Figure 7C1, arrowheads) and part of the neuronal cytoplasm is clearly missing (open arrows in Figure 7C2) beneath the microglia cell body.

**Figure 8:**
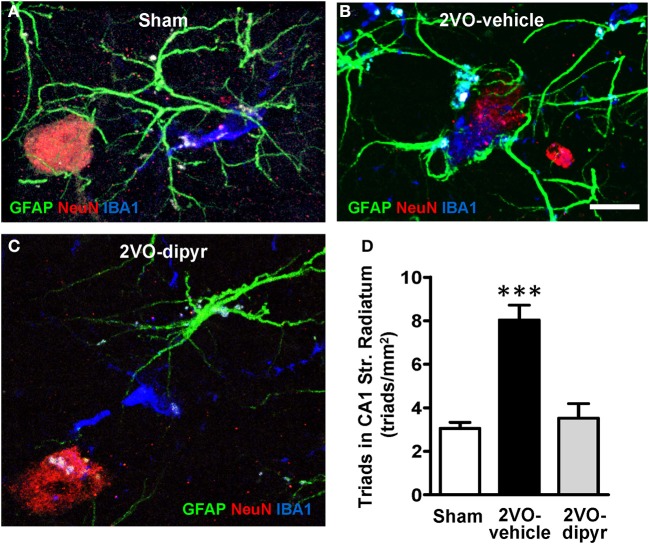
**Quantitative analysis of triads in CA1 Str. Radiatum**. Triple immunostaining of GFAP (green), NeuN (red), and IBA1 (blue) in CA1 Str. Radiatum of a sham **(A)**, a 2VO-vehicle **(B)**, and a 2VO-dipyridamole **(C)** rat. **(A)** Confocal 3D rendering of triple immunostaining in CA1 Str. Radiatum of a sham rat obtained stacking 35 consecutive confocal z scans (0.3 μm each, total thickness 10.5 μm acquired at 5.4 μm depth into the slice). **(B)** Confocal 3D rendering of triple immunostaining in CA1 Str. Radiatum of a 2VO-vehicle rat obtained stacking 49 consecutive confocal z scans (0.3 μm each, total thickness 14.7 μm). **(C)** Confocal 3D rendering of triple immunostaining in CA1 Str. Radiatum of a 2VO-dypiridamole rat obtained stacking 30 consecutive confocal z scans (0.3 μm each, total thickness 9 μm acquired at 6.9 μm depth into the slice). Note the absence of a direct interplay among neurons, astrocytes and microglia in sham **(A)** and 2VO-dipyr **(B)**. Scale bar in **A–C**: 15 μm. **(D)** Quantitative analysis of triads in CA1 Str. Radiatum of sham (*n* = 4), 2VO-vehicle (*n* = 5), and 2VO-dipyridamole (*n* = 4) rats (triads/mm^2^, mean ± s.e.m.; ^***^P < 0.01 vs. sham and 2VO-dipyr rats; One-Way ANOVA and Newman–Keuls Multiple Comparison Test).

Further representative images of neurons-astrocytes-microglia triads from sham, 2VO-dipyridamole and 2VO-vehicle rats are shown in Figures 8A–C. Quantitative analysis of the neuron-astrocytes-microglia triads (as defined in Methodological considerations) was performed in ROI of CA1 Str. Radiatum of shams (*n* = 4), 2VO-vehicle (*n* = 5), and 2VO-dipyridamole rats (*n* = 4). Results show that triads significantly increased in Str. Radiatum of 2VO-vehicle rats [triad/mm^2^; +167% vs. sham rats; One-Way ANOVA *F*_(2, 10)_ = 23.01; *P* = 0.0003; ^***^P < 0.001 vs. two other groups, Newman–Keuls Multiple Comparison Test]. Dipyridamole significantly prevented this effect (+12% vs. sham rats; n.s.).

### Quantification of apoptotic neurons in CA1 Str. Pyramidale

**Figure 9:**
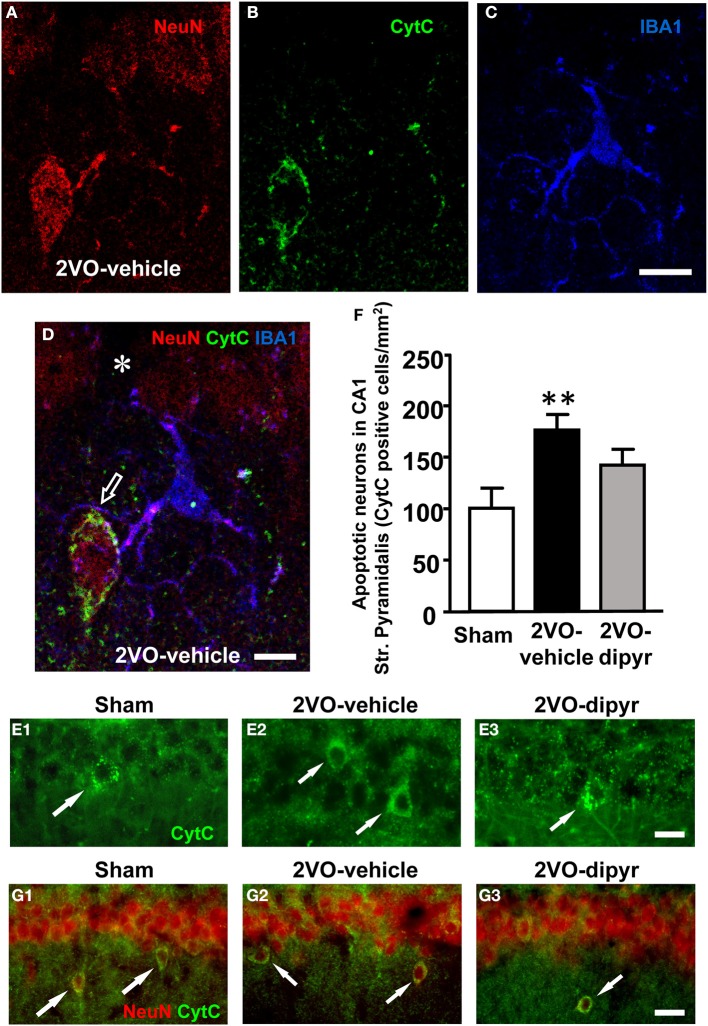
**Immunofluorescence of CytC and quantitative analysis of CytC positive neurons in CA1 Str. Pyramidale. (A–D)** Confocal immunostaining of neurons (NeuN, red), Cyt C (green), and microglia (IBA1, blue) in CA1 Str. Pyramidale and Str. Radiatum. Panels **(A–D)** show a cell “sub-slice,” obtained stacking 2 confocal z scans (0.3 μm each, total thickness 0.6 μm acquired at 1.8 μm depth into the neuron). Scale bar: 15 μm. (**D)** Merge of the three above images showing the colocalization of CytC with NeuN in the cytoplasm of an ectopic neuron and the close proximity of a microglia cell projecting its branches to surround the neuron (open arrow). The asterisk in the pyramidal cell layer shows an empty spot possibly in correspondence to the place where the neuron was previously located. Scale bar: 8 μm. **(E1–E3)** Representative images showing CytC positive neurons in the pyramidal cell layer of the three experimental groups (arrows). Scale bar: 10 μm. **(F)** Quantitative analysis of CytC positive neurons in Str. Pyramidale of sham (white column, *n* = 10), 2VO-vehicle (black column, *n* = 12), and 2VO-dipyridamole (gray column, *n* = 10) rats (CytC positive neurons/mm^2^, mean ± s.e.m.; ^**^P < 0.01 vs. sham; One-Way ANOVA and Newman–Keuls Multiple Comparison Test). Quantification was performed blind by two researchers in the region of interest (ROI) of CA1 Str. Pyramidale (represented in **E1–E3**) and results were averaged. ROIs were calculated in mm^2^ and the counts of CytC positive cells were expressed as neurons/mm^2^. **(G1–G3)** Representative images showing that ectopic neurons are CytC positive in the CA1 Str. Radiatum of the three experimental groups (arrows). Scale bar: 15 μm.

It was previously demonstrated, in a similar animal model of chronic hypoperfusion, that CA1 pyramidal neurons undergo apoptotic death between 2 and 25 weeks after the onset of 2VO (Bennett et al., 1998). Therefore, in order to verify in our model whether neurons undergoing phagocytosis by microglia were apoptotic, hippocampal slices from sham, 2VO-vehicle, and 2VO-dipyridamole rats were triple immunostained with anti-NeuN for neurons (red), with anti-CytC, a marker for apoptosis (green), and with anti-IBA1 for microglia (blue) (Figures 9A–D). The presence of a diffuse CytC immunostaining in the cytoplasm is an indication that the cell is undergoing apoptosis (Suen et al., 2008). Indeed, it has been demonstrated that at late stages of apoptosis CytC, following complete release from the mitochondria, becomes visible in the cytoplasm (Suen et al., 2008). Figure 9A shows immunostaining of neurons, Figure 9B of CytC, and Figure 9C of microglia (IBA1 positive). The merge of the three images is shown in Figure 9D. Colocalization of anti-CytC with NeuN was present in the cytoplasm of some CA1 pyramidal neurons and in ectopic neurons (Figure 9D). Some of these apoptotic CytC positive neurons were in close contact with microglia cells possibly in the act of phagocitosing the dying neuron (Figure 9D). From Figure 9D it is interesting to note that the CytC immunopositive ectopic neuron is located within 20 μm from the pyramidal cell layer. An empty spot is clearly visible in the pyramidal cell layer (asterisk), possibly in correspondence to the place where the neuron was previously located. All CytC positive pyramidal cells were neurons since they were also NeuN positive. CytC positive neurons in Str. Pyramidalys, evidenced in Figures 9E1–E3 by the arrows, were counted. Quantitative analysis shown in Figure 9F demonstrate that CytC positive cells were significantly more numerous in 2VO-vehicle rats (+76%; *n* = 10) than in sham rats (*n* = 12). Dypiridamole (*n* = 10) significantly reverted this effect, although not completely [One-Way ANOVA *F*_(2, 29)_ = 5.294; *P* = 0.011; ^**^P < 0.01 vs. shams, Newman–Keuls Multiple Comparison Test]. Arrows in panels 9G1–G3 show that ectopic neurons (NeuN positive, red) are also CytC positive (green), indicating these ectopic neurons are apoptotic. About 90% of ectopic neurons are CytC positive.

## Discussion

Brain chronic hypoperfusion, caused in the elderly by partial carotid occlusion, is a progressive, dynamic process that causes multiple alterations as ischemic conditions persist, leading to neurodegeneration (Farkas et al., 2007; Ozacmak et al., 2007) and vascular dementia (Chmayssani et al., 2007).

In this research we used a model of brain chronic hypoperfusion obtained with the permanent 2VO (bilateral common carotid artery occlusion) in the rat (Sarti et al., 2002a,b; Farkas et al., 2007). As outlined by Farkas et al. (2007) the rat represents a suitable animal species for this purpose since, after the bilateral carotid artery occlusion, the complete circle of Willis affords reduced, although continuous, blood flow to the brain (Otori et al., 2003). Therefore, the rat 2VO model is useful to investigate the long-term effects of chronic cerebral hypoperfusion, without exacerbating factors such as hypotension or hypoxia, and reperfusion injury (Farkas et al., 2007). Furthermore, the cerebral hypoperfusion is global and the damage to the nervous tissue is chronic but less dramatic than in focal ischemic models, and there are no obvious signs of motor dysfunction or seizures. Nevertheless, the CA1 region of the hippocampus results particularly vulnerable to the 2VO-related pattern of hypoperfusion and metabolic changes, leading to failure of neuronal signaling, and to learning and memory disturbances in hippocampally-based forms of memory (De Jong et al., 1999; Liu et al., 2005; Farkas et al., 2006; Melani et al., 2010).

Immunohistochemistry for NeuN and confocal microscopy analysis revealed the presence of significantly more numerous “ectopic” pyramidal neurons in the CA1 Str. Radiatum of 2VO rats, localized just outside the CA1 layer, likely deriving from CA1 Str. Pyramidale. This phenomenon may represent a sign of alteration and damage of the hippocampal CA1 Pyramidal neurons. Indeed, it had been previously demonstrated that 4 weeks after 2VO significant neuronal damage was observed in CA1 hippocampal region (Ohtaki et al., 2006). The neurons we defined as ectopic neurons had a pyramidal shape, typical of pyramidal neurons, different from other neuronal cell types, and were located within 100 μm from the Str. Pyramidale. As reported by Klausberger and Somogyi (2008), most of the GABAergic hippocampal inhibitory interneurons are located in the Str. Radiatum far from the Str. Pyramidale, and in the Str. Lacunosum Molecularis, therefore most of ectopic neurons were not GABAergic interneurons.

Using the triple immunostaining of neurons, astrocytes and microglia we demonstrated that in the Str. Radiatum of 2VO-vehicle rats many ectopic neurons were surrounded by astrocyte branches and microglia to form the so-called triads. Triads were significantly more numerous in the Str. Radiatum of 2VO-vehicle rats than in the two other groups. Using immunostaining with a marker for apoptosis we demonstrated that in CA1 of 2VO-vehicle rats many ectopic pyramidal neurons were apoptotic. With the digital sub-slicing technique developed in our laboratory (Cerbai et al., 2012), we demonstrated that many astrocyte branches not only surrounded pyramidal ectopic neurons, but also intermingled and penetrated the neuronal cytoplasm, possibly to fragment the neuronal cell body and to give rise to cellular debris. We thus hypothesized that the fragmentation of ectopic pyramidal neurons might be a consequence of the apoptotic process which these neurons were undertaking because of the hypoxic state of the tissue after bilateral common carotid occlusion. Fragmentation of apoptotic pyramidal neurons might be triggered or accelerated by astrocyte branches infiltrating the neuronal body, thus forming neuronal debris ready for phagocytosis by microglia, as also demonstrated in other models of neurodegeneration (Polazzi and Monti, 2010; Cerbai et al., 2012; Huizinga et al., 2012).

Astrogliosis is generally considered a late-emerging event in the hippocampus during chronic cerebral hypoperfusion (Pappas et al., 1996; Farkas et al., 2004, 2006, 2007; Schmidt-Kastner et al., 2005). Consistently, in our hands no sign of astrocyte proliferation was evident. Nevertheless, our results show that in CA1 of 2VO-vehicle rats, astrocytes were in a reactive state. Indeed, astrocytes exhibited hypertrophic, interdigitated and frequently overlapping processes, particularly in the surroundings of ectopic neurons. Furthermore, in some instances we found that several astrocytes surrounded ectopic neurons and formed a “micro scar” around them. These events were consistently less frequent or absent in sham and 2VO-dipyridamole rats. It has been reported that reactive gliosis is a response of astrocytes to a variety of brain insults, characterized by hypertrophy of the cell bodies and processes, and increased GFAP expression (Ridet et al., 1997; Barreto et al., 2011). As reported by Sofroniew (2009; Sofroniew and Vinters, 2010), reactive astrogliosis is neither an all-or-none response, nor a single uniform process, nor always synonymous with scar formation. In moderate forms of reactive astrogliosis, astrocyte proliferation is limited, astrocytes occupy contiguous non-overlapping domains (Bushong et al., 2002) but have hypertrophic bodies and processes (Wilhelmsson et al., 2006). At its extreme level of activation, reactive astrogliosis involves scar formation with newly proliferated cells with overlapping processes. It is generally believed that reactive astrogliosis and scar formation protect neurons and tissue, restricting the spread of inflammation and preserving brain functionality (Sofroniew, 2009). Nevertheless, increasing evidence indicates that dysfunctions of the process of reactive astrogliosis and scar formation potentially contributes to, or is the primary cause of, neurodegenerative mechanisms (Sofroniew, 2009). Our hypothesis is that the phenomenon of infiltration of astrocytes branches inside the body of the apoptotic-ectopic pyramidal neurons to trigger their disgregation might be a dysfunction or an abnormality of the process of reactive astrogliosis and could have the potential to contribute to the pathophysiological mechanisms of brain chronic ischemia.

The model of brain chronic ischemia can be subdivided in three phases: an acute phase (2–3 days) immediately after the start of occlusion, characterized by hypoxia, shear stress, production of reactive oxygen species (ROS) (Carden and Granger, 2000; Eltzschig and Carmeliet, 2011), and reduction of the nitric oxide (NO) bioavailability (Yilmaz and Granger, 2010). This phase is followed by a chronic phase (several weeks), and a restitution phase in which the tissue undergoes compensatory and adaptive mechanisms (Farkas et al., 2007; Dirnagl, 2012). As reported by Iadecola and Anrather (2011), post-ischemic insult is a self-limiting process that eventually subsides and prepares the terrain for the structural and functional reorganization of the injured brain. The factors governing the restitution phase and the reestablishment of tissue homeostasis are still poorly understood. Many evidences indicate that the restitution phase is orchestrated by the interplay of numerous processes and mediators (Spite and Serhan, 2010), including removal of dying/apoptotic cells, development of an anti-inflammatory milieu, neurogenesis, and generation of pro-survival factors stimulating tissue reconstruction and repair (Nathan and Ding, 2010; Spite and Serhan, 2010). The hippocampal slices used in our experiments were obtained from animals 3 months after bilateral common carotid occlusion, a sufficient time-lapse for the tissue to undergo all the aforementioned adaptive processes of the restitution phase of brain chronic ischemia.

Despite the increase of apoptotic neurons, of ectopic neurons and of neuronal debris in hippocampal Str. Radiatum of 2VO-vehicle rats, CA1 pyramidal neurons did not decrease in comparison to sham rats, confirming previous reports that demonstrated no loss of CA1 pyramidal neurons with age (Gallagher et al., 1996). This effect could depend upon increased neurogenesis during the restitution phase of brain chronic ischemia, as shown *in vivo* (Farkas et al., 2007; Dirnagl, 2012) and in an acute *in vitro* model of hypoxia on hippocampal slices (Maraula et al., 2013). Newly generated neurons migrate from the subgranular zone (SGZ) of the Dentate Gyrus (DG) to integrate the apoptotic neurons in the CA1 pyramidal layer. Upregulation of neurogenesis after an ischemic insult in hippocampal DG has been attributed to cell death in the entorhinal cortex or in the CA1 region (Bernabeu and Sharp, 2000).

Fluorescent immunostaining of the “triad” revealed that reactive or amoeboid (Beynon and Walker, 2012) microglia cells were phagocytosing ectopic pyramidal neurons in CA1 Str. Radiatum of 2VO-vehicle rats. These phagocytic events were consistently less frequent or absent in sham and 2VO-dipyridamole rats. Using morphological analysis of IBA1 immunostained microglia it was possible to evaluate their state of activation and to distinguish between resting and reactive microglia (Stence et al., 2001; Nelson et al., 2002; Rezaie et al., 2002; Herber et al., 2006; Miller and Streit, 2007). The quantification of resting, reactive and total microglia cells revealed that in 2VO-vehicle rats there was a significant decrease in total microglia in comparison to sham rats and dipyridamole reverted this effect. It is not surprising that the number of triads increases without significant increase of microglia and/or astrocytes because triad formation does not depend on the number of astrocytes or microglia, separately, but depends on the close interaction and interplay among neurons, astrocytes and microglia (usually phagocytic microglia). It is therefore reasonable that the number of triads increases even in the absence of any increase of the number of cells. The percentage of resting/reactive microglia was not altered by the ischemic conditions, and indeed sham, 2VO-vehicle and 2VO-dipyridamole rats, all had about 60% of resting and 40% of reactive microglia. These results on microglia population could be interpreted as follows: the decrease of total microglia may depend on the anti-inflammatory milieu promoted by the release of anti-inflammatory cytokines during the restitution phase of brain chronic hypoperfusion (Spite and Serhan, 2010). The production of these cytokines is promoted by phagocytosis, occurs in concert with the removal of dead cells (Nathan and Ding, 2010), and may have both neuroprotective properties and anti-inflammatory effects (Liesz et al., 2009). Therefore, post-ischemic production of cytokines and/or endogenous glucocorticoids (Farkas et al., 2002) can facilitate tissue repair by promoting the resolution of inflammation and exerting direct cytoprotective effects on surviving cells in the ischemic area. The effect of the anti-inflammatory cytokines could also explain the lack of modification in the percentage of resting/reactive microglia in 2VO-vehicle rats in comparison to sham rats. Indeed, the anti-inflammatory milieu could promote the recovery of the steady state level of activation of microglia cells that we found in shams. It would be of interest to investigate the presence of an upregulation of reactive microglia in the acute phase of brain chronic hypoperfusion, taking hippocampal slices at earlier times after 2VO-vehicle.

It had previously been demonstrated that treatment with dipyridamole significantly restores Morris water maze memory 90 days after 2VO (Melani et al., 2010). Dipyridamole was originally introduced in 1959 as an antianginal medication (Picano, 1989), and it has been shown to have beneficial effects in therapy for secondary stroke prevention (Halkes et al., 2006). The potent inhibition of platelet activation (Heptinstall et al., 1986) and the reduction of thrombi formation *in vivo* (Elkeles et al., 1968) accounts for this beneficial effects (Halkes et al., 2006). Dipyridamole has been tested in our model of brain chronic hypoperfusion to further evaluate its protective role on the hippocampal CA1 region during the ischemic insult. It has been demonstrated that cerebral blood flow is still significantly lower than the control values 4 weeks after 2VO induction (Schmidt-Kastner et al., 2001; Otori et al., 2003; Tomimoto et al., 2003), returning to control values only 6 months after occlusion (Choy et al., 2006). In arteries, chronic decrease of blood flow induces inward remodeling (Buus et al., 2001; Loufrani et al., 2002), such as enlargement and tortuosity of the basilar artery in response to permanent 2VO-vehicle (Schmidt-Kastner et al., 2005; Kantor et al., 2007) and this effect can take place in a period as short as 2 days (Buus et al., 2001). The increases of extracellular adenosine (Figueredo et al., 1999), together with increased VEGF production (Ernens et al., 2010), and the potentiation of the NO system (Aktas et al., 2003; Venkatesh et al., 2010) help explaining the vasodilatatory, and possibly the protecting effect of dipyridamole in this rat model of hypoperfusion. Furthermore, as reported by Venkatesh et al. (2010) dipyridamole alone in control animals has a positive effect on blood flow and angiogenesis through the nitrite/NO endocrine system. Furthermore, according to Pattillo et al. (2011), dipyridamole alone increases NO levels and decreases superoxide formation both in ischemic and non-ischemic animals. These effects indicate a positive effect of dipyridamole even in non-ischemic conditions and were commented by the authors that suggested the possible use of dipyridamole as an unique preconditioning agent that increases NO/nitrite responses (Venkatesh et al., 2010). Dipyridamole has pleiotropic pharmacological effects, such as antioxidant (Eisert, 2002; Blake, 2004; Riksen et al., 2005; Hsieh et al., 2010) and anti-inflammatory proprieties. Dipyridamole decreases the production of proinflammatory cytokines (Al Bahrani et al., 2007), chemokines (Weyrich et al., 2005), inhibits matrix metalloprotease-9 (Weyrich et al., 2005), COX-2 activity (Chen et al., 2006), and the neutrophil adhesion to endothelium (Chello et al., 1999). We hypothesize that the administration of dipyridamole during the acute phase of brain chronic hypoperfusion is responsible for the smart opposition toward the progression of the pathophysiological mechanisms of the ischemic insult. This hypothesis is sustained by the reversion of many of the effects observed in 2VO-vehicle rats by dipyridamole. Indeed, the drug reverted the significant increment of apoptotic and ectopic pyramidal neurons in hippocampal CA1 and the infiltration of astrocytes branches inside the neuronal body of ectopic pyramidal neurons. Further experiments are needed to fully understand the mechanisms of dipyridamole in rescuing the CA1 hippocampal region from the damages induced by chronic hypoperfusion.

It is important to notice that the phenomenon that astrocytes, infiltrating and bisecting neurons, may help their fragmentation was firstly demonstrated in a recent paper from our laboratory (Cerbai et al., 2012), in which we characterized the interaction neurons-astrocytes-microglia in normal brain aging and LPS-induced neurodegeneration in the rat. More recently, Re et al. (2014) demonstrated that astrocyte-motoneuron interactions are sufficient to trigger neurodegeneration in a model of Amyotrophic Lateral Sclerosis (Re et al., 2014). Therefore, this phenomenon seems to be a common mechanism underlying many neurodegenerative processes. The frequency to which it appears might depend upon, or might be the cause of, the burden and severity of neurodegeneration.

## Conclusions

Taken together, our results demonstrate the presence of neuronal damage and alteration in the interplay between neurons and glia in CA1 of 2VO-vehicle rats. The effect of dipyridamole to revert or slow the progression of the pathophysiological mechanisms of brain chronic hypoperfusion might depend upon its role as an anti-inflammatory and vasodilatory drug during the acute phase of the ischemic insult.

### Conflict of interest statement

The authors declare that the research was conducted in the absence of any commercial or financial relationships that could be construed as a potential conflict of interest.

## Supplementary material

Media File 1 **Digital 360° rotation of a 3D rendering of a neuron-astrocyte interplay showing the close contact of astrocyte branches on the neuronal cell body**. Red, neurons; green, astrocytes.
